# Preconception Hair Mercury and Serum Omega-3 Fatty Acids in Relation to Gestational Weight Gain Among Women Seeking Fertility Care

**DOI:** 10.3390/toxics13110962

**Published:** 2025-11-06

**Authors:** Han Han, Xinxiu Liang, Xilin Shen, Paige L. Williams, Tamarra James-Todd, Yazeed Allan, Roe P. Keshet, Jennifer B. Ford, Kathryn M. Rexrode, Jorge E. Chavarro, Russ Hauser, Lidia Mínguez-Alarcón

**Affiliations:** 1 Departments of Nutrition, Harvard T.H. Chan School of Public Health, Boston, MA 02115, USA; hhan@hsph.harvard.edu (H.H.); xliang@hsph.harvard.edu (X.L.); yazeedallan1@gmail.com (Y.A.); jchavarr@hsph.harvard.edu (J.E.C.); 2 Key Laboratory of Reproductive Genetics (Ministry of Education) and Department of Reproductive Endocrinology, Women’s Hospital, Zhejiang University School of Medicine, Hangzhou 310006, China; xilinshen@hsph.harvard.edu; 3 Departments of Biostatistics, Harvard T.H. Chan School of Public Health, Boston, MA 02115, USA; paige@sdac.harvard.edu; 4 Department of Epidemiology, Harvard T.H. Chan School of Public Health, Boston, MA 02115, USA; tjtodd@hsph.harvard.edu (T.J.-T.); rhauser@hsph.harvard.edu (R.H.); 5 Department of Environmental Health, Harvard T.H. Chan School of Public Health, Boston, MA 02115, USA; rkeshet@hsph.harvard.edu (R.P.K.);; 6 Division of Women’s Health, Department of Medicine, Brigham and Women’s Hospital and Harvard Medical School, Boston, MA 02115, USA; krexrode@bwh.harvard.edu; 7 Channing Division of Network Medicine, Department of Medicine, Brigham and Women’s Hospital and Harvard Medical School, Boston, MA 02115, USA; 8 Department of Obstetrics, Gynecology and Reproductive Biology, Harvard Medical School, Boston, MA 02115, USA

**Keywords:** mercury, eicosapentaenoic acid, docosahexaenoic acid, omega-3, gestational weight gain

## Abstract

Few studies have prospectively examined the joint effect of mercury (Hg) and omega-3 long-chain polyunsaturated fatty acids on gestational weight gain (GWG). This exploratory study included 120 women from the Environment and Reproductive Health (EARTH) study with preconception measurements of hair Hg and serum eicosapentaenoic acid (EPA) and docosahexaenoic acid (DHA), followed through pregnancy. Linear regression was used to examine associations between hair Hg and GWG, while logistic regression estimated the odds of inadequate GWG based on National Academy of Medicine recommendations. In unadjusted models, higher hair Hg (≥1 vs. <1 ppm) was associated with lower GWG (β = −1.89; 95% CI: −3.70, −0.08) and increased odds of insufficient GWG (OR = 2.27; 95% CI: 1.00, 5.18). However, after multivariable adjustment including serum EPA + DHA, these associations were attenuated and became non-significant. A negative, though not statistically significant, association between hair Hg and GWG was observed among women in the lowest tertile of serum EPA + DHA (≥1 vs. <1 ppm: β = −3.26; 95% CI: −7.69, 1.17), whereas no such association was observed among those in higher tertiles (β [95% CI] = 0.44 [−4.21, 5.09] and −1.05 [−4.13, 2.02], respectively). Our findings suggest that the association between preconception Hg exposure and insufficient GWG may differ by serum EPA + DHA, but results require confirmation in cohorts with larger sample sizes.

## 1. Introduction

Achieving optimal gestational weight gain (GWG) is essential for maternal and offspring health. Compared with GWG within the ranges recommended by the National Academy of Medicine (NAM) [1], insufficient GWG has been associated with higher risks of small for gestational age, preterm birth, and maternal cardiovascular disease, whereas excessive GWG is linked to increased risks of large for gestational age, macrosomia, and long-term maternal cardiometabolic morbidity and mortality [2,3,4,5]. However, the prevalence of suboptimal GWG remains high in the US, with over 20% of pregnant women gaining insufficient weight and more than 50% gaining excessively [6]. Therefore, identifying modifiable environmental and dietary factors to inform interventions aimed at gestational weight management is a pressing public health priority.

Mercury (Hg) is a persistent neurotoxin, and its organic form, methylmercury (MeHg), is among the most commonly encountered in the US, primarily through dietary sources such as fish and other seafood [7]. The neurological toxicity of MeHg exposure is well established, and accumulating evidence indicates that prenatal exposure impairs offspring neurodevelopment through maternal-fetal transfer; however, its impact on weight regulation remains unclear [8]. Several animal studies have reported reduced body weight following high-dose MeHg exposure [9,10,11], while human studies have shown positive [12,13], negative [14,15,16], or null [17] associations between Hg biomarkers and obesity in the general population. However, direct evidence linking Hg exposure to weight change during pregnancy remains limited. Given that certain fish and seafood are also rich in beneficial omega-3 (n-3) long-chain polyunsaturated fatty acids (LC-PUFAs) [18], it is plausible that n-3 LC-PUFAs may mitigate the adverse effects of MeHg, potentially contributing to inconsistent findings across studies. Therefore, the trade-off between the risks of MeHg exposure and the benefits of n-3 LC-PUFA intake on GWG, as well as the underlying biological mechanisms, warrants further investigation.

To fill these knowledge gaps, we prospectively examined the joint association between hair Hg concentrations and serum n-3 LC-PUFA levels in relation to GWG among women who underwent in vitro fertilization (IVF) in the Environment and Reproductive Health (EARTH) study.

## 2. Materials and Methods

### 2.1. Study Population

The EARTH study is a prospective preconception study designed to evaluate the environmental, dietary, and lifestyle determinants of fertility [19]. The cohort recruited women and men seeking fertility evaluation and medical treatment at the Massachusetts General Hospital (MGH) Fertility Center. Between 2004 and 2019, 991 female participants aged 18–45 years were enrolled, and 699 became pregnant. Of these, 146 provided hair samples for Hg analysis and had serum n-3 LC-PUFA biomarker data. After excluding 25 participants with missing gestational weight information at or beyond the first trimester and one with a triplet birth, 120 women with singleton or twin births were included in this prospective analysis. The EARTH study was approved by the Institutional Review Boards of MGH and the Harvard T.H. Chan School of Public Health. Informed consent was obtained from all participants.

### 2.2. Assessment of Hair Hg

Hair Hg was selected as the preferred biomarker because it primarily comprises methylmercury (MeHg) and reflects exposure over months [20]. Hair samples were voluntarily provided by female participants after enrollment and mailed in envelopes to study staff at their convenience during the study period. Hair samples were first cleaned by sonication in a 1% Triton X-100 solution for 15 min to remove extraneous contaminants, then rinsed with distilled deionized water and dried at 60 °C for 48 h. Total Hg concentration in hair was measured in parts per million (ppm) using 0.02 g of the proximal 2 cm segment of hair, analyzed with a Direct Mercury Analyzer 80 (Milestone Inc., Monroe, CT, USA) using a matrix-matched calibration curve. Since hair grows approximately 1 cm per month [21], the 2 cm segment reflects roughly two months of exposure. Therefore, the current study included only hair samples collected before or within one month after the first-trimester weight measurement to ensure that the captured Hg exposure preceded the study outcome. Certified reference material GBW 07601 (human hair; Institute of Geophysical and Geochemical Exploration, China), containing 360 ppm Hg, was used as the quality control standard. Percent recovery ranged from 90% to 110%, and the percent difference between duplicate samples at the same time point was below 10%. The limit of detection (LOD) for Hg was 0.01 ppm.

### 2.3. Assessment of Serum Omega-3 Fatty Acids

In our study, most female participants underwent multiple cycles of IVF to achieve pregnancy and provided blood samples accordingly. For each participant, the blood sample from the first fresh cycle was selected for quantifying fatty acids including EPA and DHA. Serum samples were aliquoted, frozen, and stored at −80 °C until transfer to the Biomarker Research Laboratory at the Harvard T.H. Chan School of Public Health (Boston, MA, USA) for analysis. As previously described [22], fatty acids were extracted and transmethylated with methanol and sulfuric acid [23,24], and the resulting fatty acid methyl esters were re-dissolved in iso-octane and quantified using capillary gas–liquid chromatography. External validation was performed through participation in programs organized by the American Oil Chemists’ Society and the National Institute of Standards and Technology. Coefficients of variation (CVs) for fatty acids were monitored using pooled control samples included in each extraction and analysis batch. Mean CVs across 16 pairs of control samples were 7.3% for EPA and 7.2% for DHA. Given that fish and seafood are primary dietary sources of both EPA and DHA, we summed EPA and DHA (hereafter referred to as EPA + DHA) for subsequent analysis.

### 2.4. Assessment of Maternal Weight

Participants’ weight and height were measured by trained study staff at enrollment using instruments calibrated for clinical use. Maternal gestational weights measured during routine prenatal visits and corresponding gestational weeks were abstracted from electronic medical records, including measurements from the first trimester (median gestational week = 10.1 weeks) and at delivery (median gestational week = 38.4 weeks). Given that most participants underwent IVF cycles of varying durations prior to conception, which spanned several months to years, weight measured during the first trimester, rather than weight measured at study enrollment, was considered a proxy for pre-pregnancy weight. Total GWG was calculated as weight at delivery minus weight in the first trimester, and pre-pregnancy BMI was calculated as first-trimester weight (in kilograms [kg]) divided by height (in meters [m]) squared. According to the 2009 NAM guidelines [1], which provide BMI-specific GWG recommendations for singleton pregnancies and provisional ranges for twin pregnancies, we defined three binary outcome variables indicating whether GWG was below, within, or above the NAM-recommended ranges (Table S1). We further adjusted for the length of gestation to reduce the risk of potential misclassification of GWG [25].

### 2.5. Covariates

Information on sociodemographic factors, lifestyle behaviors, and medical history was self-reported through questionnaires administered at enrollment, while clinical variables were abstracted from electronic medical records. Infertility diagnoses were made by physicians based on criteria established by the Society of Assisted Reproductive Technology (SART).

### 2.6. Statistical Analysis

The distributions of demographic and reproductive characteristics, as well as biomarkers, were summarized as counts (percentages) or medians with interquartile ranges (IQRs) for the overall study population and subgroups defined by hair Hg concentrations. Subgroups were classified using the US Environmental Protection Agency reference level of 1 ppm in hair [26]. Missing data were imputed using reference values for categorical covariates (i.e., missing maternal education was imputed as the “less than graduate degree” group) and median values for continuous covariates (i.e., gestational ages). The missing rate is shown in Table 1. Spearman correlation coefficients (r_s_) were calculated to examine the relationship between hair Hg concentrations and serum n-3 LC-PUFA biomarkers (i.e., EPA and DHA). Biomarker levels were log-transformed before subsequent analyses due to their right-skewed distributions.

To examine the association between hair Hg concentrations and GWG, we fit linear regression models with continuous GWG (in kg) as the outcome and categorical exposure variables as predictors. The β estimates with 95% confidence intervals (CIs) were reported for each category relative to the reference group. Hair Hg concentrations were categorized into tertiles (with the lowest tertile as the reference) and dichotomized based on the US Environmental Protection Agency reference level (<1 ppm vs. ≥1 ppm). Median values of hair Hg within each tertile were also modeled as continuous variables to evaluate linear trends. Multivariable logistic regression models were used to estimate odds ratios (ORs) and 95% CIs for insufficient or excessive GWG, defined as outside the NAM-recommended range. Estimates from both unadjusted (Model 1) and adjusted models (Models 2 and 3) were reported. Covariates were selected based on prior knowledge of their potential associations with both the exposure and the outcome. In Model 2, we adjusted for age (years), ancestry (White or other), maternal education (graduate degree or less), pre-pregnancy BMI (kg/m^2^), smoking status (ever or never), total physical activity (hours/week), infertility diagnosis at enrollment (female factor, male factor, or unexplained), plurality (singleton or twins), gestational age at first-trimester weight measurement (weeks), and gestational age at delivery (weeks). Since Hg and EPA + DHA share common exposure sources (e.g., fish and seafood), we conducted three sets of analyses to try to identify their independent and joint effects. First, we fit an additional multivariable model (Model 3) that included serum EPA + DHA levels as an additional covariate, in addition to the variables included in Model 2. Second, we conducted a stratified analysis by serum EPA + DHA tertiles to examine the association between Hg and GWG. Third, we modeled the joint association of hair Hg and serum EPA + DHA with GWG by categorizing women into six groups based on dichotomized Hg (<1 vs. ≥1 ppm) and EPA + DHA tertiles or into nine groups based on tertiles of both biomarkers. For stratified and joint analyses, models were adjusted for the same covariates as Model 2.

To examine other potential effect modification, we conducted analyses of the relation between Hg and GWG stratified by pre-pregnancy BMI (<25 or ≥25 kg/m^2^), plurality (singleton vs. twins), and maternal educational attainment (graduate degree or less). These stratifying variables were selected based on prior evidence of their relevance to weight gain during pregnancy [1,27]. The stratified models were adjusted for the same covariates as Model 3, excluding the stratifying variable. We conducted several sensitivity analyses to assess the robustness of our findings. First, given that the NAM guidelines provide only provisional GWG ranges for twin pregnancies, we restricted the primary analyses to women with singleton pregnancies. Second, instead of including both gestational age at delivery and gestational age at first-trimester weight measurement in the model, we adjusted for gestational length, defined as the difference between the two, to examine the association between Hg and GWG. Third, we excluded participants with missing maternal education data (n = 12) and repeated the corresponding stratified analysis. A two-sided *p* value < 0.05 was considered statistically significant. All statistical analyses were performed in R version 4.3.0.

## 3. Results

Our study included 120 women with a median age of 35.0 (IQR: 33.0, 39.0) years (Table 1). Participants in the current study were predominantly White (80.0%) and had a median GWG of 13.6 kg, with 29.2% classified as inadequate, 37.5% as appropriate, and 33.3% as excessive based on the NAM-recommended ranges. Compared with those with lower hair Hg concentrations (≤1 ppm), participants with a hair Hg concentration of >1 ppm were older (median 38.0 vs. 34.0 years), had higher education attainment (71.8% vs. 46.9% with a graduate degree), higher serum EPA + DHA levels (median 3.7% vs. 2.8% of total serum fatty acids), and lower GWG (median 12.7 vs. 14.5 kg). Hair Hg concentrations showed a moderate correlation with serum EPA + DHA levels (Spearman correlation coefficient, r_s_ = 0.30; Table S2), especially for DHA (r_s_ = 0.35).

In the unadjusted models (Table 2), higher hair Hg concentrations (≥1 vs. <1 ppm) were associated with lower GWG (β [95% CI]: −1.89 [−3.70, −0.08]; *p* = 0.04). Regarding the probability of achieving the NAM-recommended GWG range (Table S3), participants with higher hair Hg concentrations (≥1 vs. <1 ppm) had increased odds of insufficient GWG (OR: 2.27; 95% CI: 1.00, 5.18; *p* = 0.05), but not of excessive GWG (OR: 0.59; 95% CI: 0.24, 1.34]; *p* = 0.22). However, these associations were attenuated and no longer statistically significant after adjusting for potential confounders, including serum EPA + DHA levels (Table 2 and Table S3). The results remained consistent when we restricted to women with singleton pregnancy (Table S4) or adjusted for gestational length (median gestational length = 28.0 weeks) instead of including both gestational age at delivery and at the first-trimester weight measurement in the model.

**Figure 1:**
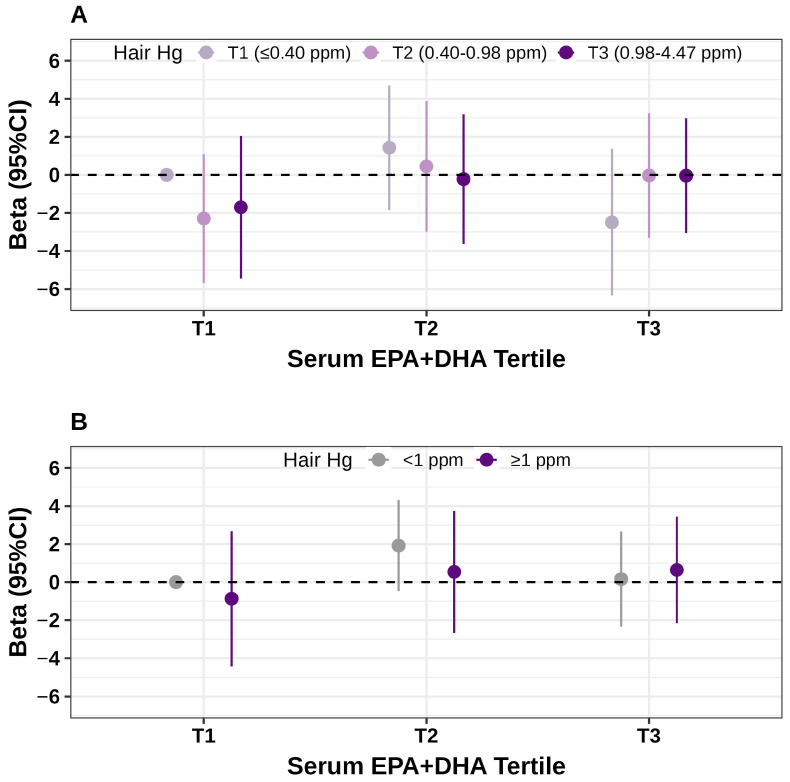
Joint associations of hair Hg and serum EPA + DHA with continuous GWG. Linear regression models were adjusted for age (years), ancestry (White or other), educational attainment (graduate degree or less), pre-pregnancy body mass index (kg/m^2^), smoking status (ever or never), total physical activity (hours/week), infertility diagnosis at enrollment (female factor, male factor, or unexplained), plurality (singleton or twins), gestational age at first-trimester weight measurement, and at delivery (weeks). (**A**) Exposure groups defined by tertiles of hair Hg and serum EPA + DHA. (**B**) Exposure groups defined by dichotomized Hg (<1 vs. ≥1 ppm) and EPA + DHA tertiles. The lowest group for both exposures served as the reference. Abbreviations: DHA, docosahexaenoic acid; EPA, eicosapentaenoic acid; Hg, mercury; GWG, gestational weight gain.

A negative, though not statistically significant, association between hair Hg concentration and GWG was observed among women with lower serum EPA + DHA levels, but not among those with higher levels (Table 3). When comparing high Hg concentration group (≥1 ppm) with the lower group (<1 ppm), the multivariable-adjusted β estimates for GWG were −3.26 (95% CI: −7.69, 1.17) in the lowest tertile, 0.44 (−4.21, 5.09) in the middle tertile, and −1.05 (95% CI: −4.13, 2.02) in the highest tertile of serum EPA + DHA levels. A similar trend was observed when hair Hg concentrations were analyzed by tertiles. In the joint analysis, women with higher hair Hg and low serum EPA + DHA levels appeared to have lower GWG compared with those with low levels of both exposures; however, these associations did not reach statistical significance (Figure 1 and Table S5).

We observed no associations between hair Hg concentrations and GWG across subgroups defined by pre-pregnancy BMI or plurality (Table 3). When stratified by educational attainment, no association between hair Hg concentrations and GWG was observed among participants with a graduate degree, either before or after adjustment for serum EPA + DHA levels. However, among those without a graduate degree or with missing maternal education data, the inverse association became more pronounced after adjusting for serum EPA + DHA levels. When comparing the highest and lowest tertiles of hair Hg concentrations, the multivariable-adjusted β estimates for GWG were −1.77 (95% CI: −5.62, 2.09; *P* for trend = 0.29; Table S6), which strengthened to −3.24 (95% CI: −7.20, 0.72; *P* for trend = 0.08; Table 3) following further adjustment for serum EPA + DHA. The observation was similar after excluding participants with missing maternal education data (Table S7).

## 4. Discussion

In this prospective pilot study of 120 pregnant women seeking fertility care, we found that higher Hg concentrations (≥1 ppm) were significantly associated with lower weight gain during pregnancy and increased unadjusted odds of insufficient GWG. However, these associations were attenuated and became non-significant after adjustment for potential confounders. A suggestive inverse association between hair Hg concentrations and GWG was observed among women in the lowest tertile of serum EPA + DHA levels, but not in the middle or highest tertiles; however, none of these associations reached statistical significance. These findings raise the possibility that the potential adverse effect of Hg exposure on insufficient GWG may be modified by maternal n-3 LC-PUFA status.

Although several animal studies have reported weight loss following Hg exposure [9,10,11], experimental evidence elucidating the underlying mechanisms remains scarce. Previous human studies on the association between Hg exposure and body weight has primarily come from observational studies in the general population, yielding inconsistent findings [12,13,14,15,16,17]. To our knowledge, this is the first study to examine the relationship between preconception Hg biomarkers and weight change during pregnancy. Among the general population, a US-based study reported no significant association between baseline toenail Hg concentrations and prospectively assessed BMI [17]. Several cross-sectional studies using data from the National Health and Nutrition Examination Survey (NHANES) [14,15,16] have reported an inverse association between blood Hg levels and obesity prevalence among non-pregnant US adults, whereas data from Asian populations suggest positive associations [12,13]. Differences in exposure levels and sources may partly explain these discrepancies. For example, Hg biomarker concentrations tend to be higher in Asian populations, possibly reflecting distinct dietary or environmental exposure pathways [13,28]. Notably, the suggestive inverse trend between hair Hg concentrations and GWG observed in our study was directionally consistent with previous findings from NHANES, although it was only evident among participants with lower serum EPA + DHA levels. However, the mean serum n-3 LC-PUFA level in our study population (3.1% of total fatty acids) was overall higher than that of pregnant women in the general US population (mean = 2.2%), [29] whose average level was comparable to that of participants in the lowest tertile of our cohort (mean = 2.2%). Additionally, it is important to note that blood Hg reflects recent exposures and captures both MeHg and inorganic Hg, whereas hair and toenail Hg are considered better indicators of long-term MeHg exposure [30]. These results highlight the need for further research on preconception MeHg exposure and GWG across diverse populations to confirm and extend our findings.

The potential effect modification by n-3 LC-PUFA has been largely overlooked in most studies examining the relationship between Hg exposure and body weight [12,13,16,17]. Among those that did account for n-3 LC-PUFA [14,15], intake was typically assessed using self-reported methods such as 24-hour dietary recalls, which are prone to both random and systematic measurement errors [31]. In this context, serum biomarkers provide an objective measure of EPA + DHA intake from various dietary sources, thereby enabling a more accurate investigation of their potential role in the association of Hg exposure and GWG. Although not statistically significant, likely due to the limited sample size, we observed a suggestive inverse association between hair Hg concentrations and GWG among participants in the lowest tertile of serum EPA + DHA, but not among those in the middle or highest tertile. This pattern suggests that the directions of association between Hg and n-3 LC-PUFA with GWG may differ, with the association between Hg and GWG potentially attenuated in the presence of high n-3 LC-PUFA. This observation supports current FDA recommendations for fish intake during pregnancy, which emphasize choosing fish that are low in MeHg and rich in healthy n-3 fats [32]. However, it should be noted that the effect of n-3 LC-PUFA on GWG remains unclear, as current evidence is limited, although a positive association direction has been observed in several intervention studies [33].

Maternal education influences food access and dietary behaviors, which are critical determinants of nutritional status during pregnancy [34]. Accordingly, women with higher education attainment may be more likely to afford and choose low-MeHg seafood, or fortified foods and supplements that provide EPA + DHA without MeHg to maximize nutritional benefits [35]. In our study, women with higher education attainment had higher hair Hg concentrations (median: 0.83 vs. 0.65 ppm) and serum EPA + DHA levels (median 3.5% vs. 2.7% of total fatty acids) but showed a weaker correlation between the two biomarkers (r_s_ = 0.18 vs. 0.36 among women without a graduate degree; Table S8), suggesting potential differences in both the dietary sources of EPA + DHA across individuals with different education attainments. Such variation may partially explain the suggestive inverse trend of hair Hg concentrations with GWG observed among women without a graduate degree after adjustment for negative confounding by EPA + DHA [36].

The strengths of our study include the use of biomarkers to assess both preconception Hg exposure and n-3 LC-PUFA intake, as well as the prospective evaluation of their potential independent and joint effect on GWG. Nevertheless, several limitations warrant discussion. First, weight information was not collected immediately before conception. Although we used the weight measured during the first trimester as a proxy and adjusted for gestational length in the model [25], potential misclassification of GWG based on NAM recommendations may still exist. Second, despite the prospective design, causal inferences cannot be established, and residual confounding remains possible given the nature of an observational study. Third, our study population comprised women undergoing IVF treatment to achieve pregnancy, which may limit the generalizability of our findings to a broader population of pregnant women. However, evidence from this subpopulation is critically needed, given the rising prevalence of subfertility in recent years [37]. Finally, the observed results should be interpreted with caution given the relatively small sample size of our study. As we observed only a suggestive association between hair Hg levels and GWG, potentially modified by n-3 LC-PUFA levels, larger studies should be conducted to further elucidate these associations in other study cohorts.

## 5. Conclusions

In this prospective exploratory study of women attending a fertility center, who overall had high n-3 LC-PUFA consumption, we observed no statistically significant association between hair Hg concentrations and GWG. However, a suggestive yet non-significant inverse association was observed among women with lower serum n-3 LC-PUFA, implying that higher n-3 LC-PUFA may attenuate the negative association between Hg exposure and GWG. Larger prospective studies in more diverse pregnant populations are warranted to confirm these findings.

## Figures and Tables

**Table 1:** Baseline characteristics by hair Hg concentrations among 120 women in the EARTH study.

	Overall	Hair Hg Concentrations ^a^	
		≤1 ppm	>1 ppm
Number of participants	120	81	39
Hair Hg concentrations, ppm	0.62 (0.33, 1.12)	0.40 (0.25, 0.62)	1.43 (1.18, 2.07)
Age, years	35.0 (33.0, 39.0)	34.0 (32.0, 38.0)	38.0 (34.0, 40.0)
White, N (%)	96 (80.0)	68 (84.0)	28 (71.8)
Educational attainment			
Less than graduate degree	42 (35.0)	34 (42.0)	8 (20.5)
Graduate degree	66 (55.0)	38 (46.9)	28 (71.8)
Missing	12 (10.0)	9 (11.1)	3 (7.7)
Ever smoked, N (%)	31 (25.8)	20 (24.7)	11 (28.2)
Primary infertility diagnosis, N (%)			
Male factor	44 (36.7)	26 (32.1)	18 (46.2)
Female factor	31 (25.8)	21 (25.9)	10 (25.6)
Unexplained	45 (37.5)	34 (42.0)	11 (28.2)
Plurality, N (%)			
Singleton	90 (75.0)	59 (72.8)	31 (79.5)
Multiples ^b^	30 (25.0)	22 (27.2)	8 (20.5)
Total physical activity, hours/week	5.0 (1.5, 9.5)	5.5 (1.50 10.0)	5.0 (1.8, 6.0)
Pre-pregnancy BMI, kg/m^2^	24.1 (21.8, 28.0)	24.7 (21.8, 28.2)	23.2 (21.7, 26.2)
Pre-pregnancy BMI categories			
Underweight (<18.5 kg/m^2^)	1 (0.8)	1 (1.2)	0 (0.0)
Normal (18.5–24.9 kg/m^2^)	67 (55.8)	41 (50.6)	26 (66.7)
Overweight (25–29.9 kg/m^2^)	37 (30.8)	29 (35.8)	8 (20.5)
Obesity (≥30 kg/m^2^)	15 (12.5)	10 (12.3)	5 (12.8)
Gestational age at first-trimester weight measurement, weeks	10.1 (9.5, 11.0)	10.1 (9.6, 11.0)	10.1 (9.4, 11.5)
Missing, N (%)	4 (3.3)	2 (2.5)	2 (5.1)
Weight at first-trimester measurement, kg	66.2 (60.3, 75.1)	68.0 (61.2, 75.7)	64.4 (59.6, 72.1)
Gestational age at delivery, weeks	38.4 (36.6, 39.7)	38.4 (36.9, 39.7)	38.0 (36.4, 39.1)
Missing, N (%)	4 (3.3)	4 (4.9)	0 (0)
Weight at delivery, kg	80.5 (73.5, 90.0)	83.0 (75.3, 91.2)	77.1 (71.0, 87.8)
Total gestational weight gain, kg	13.6 (10.9, 17.2)	14.5 (11.3, 17.7)	12.7 (10.4, 15.0)
IOM-recommended ranges for total gestational weight gain ^c^			
Below	35 (29.2)	19 (23.5)	16 (41.0)
Within	45 (37.5)	32 (39.5)	13 (33.3)
Above	40 (33.3)	30 (37.0)	10 (25.6)
Serum omega-3 fatty acids, % of total fatty acids			
EPA	0.7 (0.5, 1.3)	0.7 (0.5, 1.2)	0.8 (0.6, 1.6)
DHA	2.3 (1.8, 3.3)	2.1 (1.7, 3.0)	2.7 (2.3, 4.0)
EPA + DHA	3.0 (2.4, 4.7)	2.8 (2.3, 4.1)	3.7 (2.9, 6.1)

^a^ Categorized according to the US Environmental Protection Agency reference level for Hg exposure (≤1 ppm or >1 ppm). ^b^ Includes twin pregnancy only. ^c^ Categorized based on the NAM guidelines (see Table S1 for details). Abbreviations: BMI, body mass index; DHA, docosahexaenoic acid; EARTH, Environment and Reproductive Health; EPA, eicosapentaenoic acid; Hg, mercury; NAM, National Academy of Medicine.

**Table 2:** Associations of hair Hg concentrations with continuous GWG.

Hair Hg Concentrations, ppm	Model 1 ^a^	Model 2 ^b^	Model 3 ^c^
Tertiles (range, ppm)			
T1 (≤0.40 ppm)	Ref.	Ref.	Ref.
T2 (0.40–0.98 ppm)	−0.02 (−2.10, 2.07)	−0.76 (−2.85, 1.33)	−0.90 (−3.01, 1.22)
T3 (0.98–4.47 ppm)	−1.83 (−3.92, 0.26)	−0.62 (−2.72, 1.48)	−0.85 (−3.02, 1.32)
*P* for trend	0.09	0.53	0.41
EPA reference level			
<1 ppm	Ref.	Ref.	Ref.
≥1 ppm	−1.89 (−3.70, −0.08)	−0.40 (−2.29, 1.50)	−0.53 (−2.46, 1.40)
*p* value	0.04	0.68	0.59

Abbreviations: EPA, the US Environmental Protection Agency; GWG, gestational weight gain; Hg, mercury. ^a^ Linear regression was used to examine associations between hair Hg and continuous GWG (kg); Model 1 is the unadjusted model. ^b^ Adjusted for age (years), ancestry (White or other), educational attainment (graduate degree or less), pre-pregnancy body mass index (kg/m^2^), smoking status (ever or never), total physical activity (hours/week), infertility diagnosis at enrollment (female factor, male factor, or unexplained), plurality (singleton or twins), gestational age at first-trimester weight measurement (weeks), and gestational age at delivery (weeks). ^c^ Further adjusted for serum omega-3 long-chain polyunsaturated fatty acid levels based on Model 2.

**Table 3:** Stratified analysis of associations between hair Hg concentrations and continuous GWG ^a^.

	N	Hair Hg Concentration						
		T1 (≤0.40 ppm)	T2 (0.40–0.98 ppm)	T3 (0.98–4.47 ppm)	*P* for Trend	<1 ppm	≥1 ppm	*p* Value
**Serum EPA + DHA tertile (range ^b^)**								
T1 (1.4–2.6%)	40	Ref.	0.23 (−3.88, 4.34)	−3.22 (−7.79, 1.35)	0.25	Ref.	−3.26 (−7.69, 1.17)	0.14
T2 (2.6–3.9%)	40	Ref.	0.64 (−4.09, 5.36)	0.78 (−4.60, 6.17)	0.75	Ref.	0.44 (−4.21, 5.09)	0.85
T3 (3.9–22.8%)	40	Ref.	1.69 (−2.60, 5.98)	0.49 (−3.83, 4.82)	0.98	Ref.	−1.05 (−4.13, 2.02)	0.49
**Pre-pregnancy BMI**								
<25 kg/m^2^	68	Ref.	0.89 (−1.42, 3.20)	−0.20 (−2.30, 1.90)	0.88	Ref.	−0.57 (−2.44, 1.30)	0.55
≥25 kg/m^2^	52	Ref.	−2.65 (−6.52, 1.22)	−2.00 (−6.29, 2.29)	0.29	Ref.	−0.62 (−4.51, 3.28)	0.75
**Plurality**								
Singleton	90	Ref.	−0.29 (−2.77, 2.19)	0.09 (−2.50, 2.68)	0.97	Ref.	0.10 (−2.22, 2.43)	0.93
Twins	30	Ref.	−3.76 (−9.63, 2.10)	−2.38 (−8.41, 3.65)	0.37	Ref.	−0.49 (−5.86, 4.88)	0.85
**Educational attainment**								
Less than graduate degree	54	Ref.	−2.51 (−5.87, 0.86)	−3.24 (−7.20, 0.72)	0.08	Ref.	−1.81 (−5.50, 1.89)	0.33
Graduate degree	66	Ref.	1.39 (−1.53, 4.31)	0.34 (−2.23, 2.92)	0.77	Ref.	−0.20 (−2.51, 2.10)	0.86

^a^ Linear regression models were adjusted for age (years), ancestry (White or other), educational attainment (graduate degree or less), pre-pregnancy BMI (kg/m^2^), smoking status (ever or never), total physical activity (hours/week), infertility diagnosis at enrollment (female factor, male factor, or unexplained), plurality (singleton or twins), gestational age at first-trimester weight measurement (weeks), gestational age at delivery (weeks), and serum EPA + DHA levels, excluding the stratifying variable. ^b^ % of total fatty acids. Abbreviations: BMI, body mass index; DHA, docosahexaenoic acid; EPA, eicosapentaenoic acid; GWG, gestational weight gain; Hg, mercury.

## Data Availability

The data are not publicly available due to privacy and confidentiality reasons.

## Supplementary Materials

The following supporting information can be downloaded at: https://www.mdpi.com/article/10.3390/toxics13110962/s1, Table S1. NAM guidelines for recommended gestational weight gain; Table S2. Spearman correlations of biomarkers among 120 women in the EARTH Study; Table S3. Associations of hair Hg concentrations with the risk of not achieving the NAM-recommended GWG range; Table S4. Associations between hair Hg concentrations and GWG among women with singleton pregnancy; Table S5. Joint associations of hair Hg and serum EPA+DHA with GWG; Table S6. Stratified analysis of associations between hair Hg concentrations and GWG (without adjustment for serum EPA+DHA levels); Table S7. Associations between hair Hg concentrations and GWG among participants with lower education level; Table S8. Spearman correlations of biomarkers among women with different educational attainment.
