# Supplementary material for: Preconception Hair Mercury and Serum Omega-3 Fatty Acids in Relation to Gestational Weight Gain Among Women Seeking Fertility Care
Source: Toxics. 2025 Nov 6;13(11):962. doi: 10.3390/toxics13110962 (PMC12656212; doi:10.3390/toxics13110962)
Supplement: Supplementary file 1 [file toxics-13-00962-s001.zip › toxics-3910602-supplementary.pdf]

Supplementary Materials

Preconception Hair Mercury and Serum Omega-3 Fatty Acids in Relation to Gestational Weight Gain Among Women Seeking Fertility Care

Han Han, Xinxiu Liang, Xilin Shen, Paige L. Williams, Tamarra James-Todd, Yazeed Allan, Roe P. Keshet, Jennifer B. Ford, Kathryn M. Rexrode, Jorge E. Chavarro, Russ Hauser and Lidia Mínguez-Alarcón

Table of contents

Table S1. NAM guidelines for recommended gestational weight gain[a] 2

Table S2. Spearman correlations of biomarkers among 120 women in the EARTH Study 2

Table S3. Associations of hair Hg concentrations with the risk of not achieving the NAM-recommended GWG range 3

Table S4. Associations between hair Hg concentrations and GWG among women with singleton pregnancy (n=90) 4

Table S5. Joint associations of hair Hg and serum EPA+DHA with GWG 5

Table S6. Stratified analysis of associations between hair Hg concentrations and GWG (without adjustment for serum EPA+DHA levels)[a] 6

Table S7. Associations between hair Hg concentrations and GWG among participants with lower education level (n=42) 6

Table S8. Spearman correlations of biomarkers among women with different educational attainment .. 7

Table S1. NAM guidelines for recommended gestational weight gain[a]

Pre-pregnancy BMI	NAM-recommended ranges	
	Singleton	Twins
Underweight (<18.5 kg/m^2)	12.5-18	/
Normal (18.5-24.9 kg/m^2)	11.5-16	17-25
Overweight (25-29.9 kg/m^2)	7-11.5	14-23
Obesity (≥30 kg/m^2)	5-9	11-19

[a] NAM guideline provides recommended GWG ranges for singleton and provisional ranges for twin pregnancies.
Abbreviations: NAM, National Academy of Medicine.

Table S2. Spearman correlations of biomarkers among 120 women in the EARTH Study

	Hg	EPA	DHA	EPA+DHA
**Hg**	1	0.15	0.35	0.30
P-value		*0.10*	*<0.001*	*0.001*
**EPA**	-	1	0.63	0.79
P-value			*<0.001*	*<0.001*
**DHA**	-	-	1	0.96
P-value				*<0.001*
**EPA+DHA**	-	-	-	1

Abbreviations: DHA, docosahexaenoic acid; EPA, eicosapentaenoic acid; Hg, mercury.

Table S3. Associations of hair Hg concentrations with the risk of not achieving the NAM-recommended GWG range

Hair Hg concentrations, ppm	Model 1[a]	Model 2[b]	Model 3[c]
**GWG outside the IOM-recommended range[d]**			
Tertiles (range)			
T1 (≤0.40 ppm)	Ref.	Ref.	Ref.
T2 (0.40-0.98 ppm)	0.53 (0.21, 1.31)	0.59 (0.20, 1.75)	0.59 (0.19, 1.78)
T3 (0.98-4.47 ppm)	1.00 (0.39, 2.56)	1.04 (0.35, 3.10)	1.05 (0.34, 3.29)
P for trend	0.95	0.96	0.97
EPA reference level			
<1 ppm	Ref.	Ref.	Ref.
≥1 ppm	1.31 (0.59, 2.97)	1.26 (0.47, 3.42)	1.29 (0.47, 3.61)
P value	0.51	0.64	0.62
**GWG below the IOM-recommended range[d]**			
Tertiles (range)			
T1 (≤0.40 ppm)	Ref.	Ref.	Ref.
T2 (0.40-0.98 ppm)	0.49 (0.16, 1.40)	0.47 (0.12, 1.65)	0.51 (0.13, 1.83)
T3 (0.98-4.47 ppm)	1.56 (0.62, 3.99)	1.02 (0.34, 3.05)	1.14 (0.36, 3.65)
P for trend	0.37	0.99	0.85
EPA reference level			
<1 ppm	Ref.	Ref.	Ref.
≥1 ppm	2.27 (1.00, 5.18)	1.49 (0.54, 4.03)	1.63 (0.58, 4.65)
P value	0.05	0.44	0.35
**GWG above the IOM-recommended range[d]**			
Tertiles (range)			
T1 (≤0.40 ppm)	Ref.	Ref.	Ref.
T2 (0.40-0.98 ppm)	0.90 (0.36, 2.24)	1.25 (0.35, 4.61)	1.16 (0.31, 4.38)
T3 (0.98-4.47 ppm)	0.63 (0.24, 1.62)	1.19 (0.32, 4.51)	1.11 (0.30, 4.28)
P for trend	0.35	0.77	0.87
EPA reference level			
<1 ppm	Ref.	Ref.	Ref.
≥1 ppm	0.59 (0.24, 1.34)	0.93 (0.27, 3.08)	0.91 (0.27, 2.99)
P value	0.22	0.91	0.88

[a] Logistic regression model was used to estimate odds ratios (ORs) and 95% CIs for insufficient or excessive GWG, defined as outside the NAM-recommended range; Model 1 is the unadjusted model.
[b] Adjusted for age (years), ancestry (White or other), educational attainment (graduate degree or less), pre-pregnancy body mass index (kg/m^2), smoking status (ever or never), total physical activity (hours/week), infertility diagnosis at enrollment (female factor, male factor, or unexplained), plurality (singleton or twins), gestational age at first-trimester weight measurement (weeks), and gestational age at delivery (weeks).
[c] Further adjusted for serum omega-3 long-chain polyunsaturated fatty acid levels based on Model 2.
[d] NAM guideline provides recommended GWG ranges for singleton and provisional ranges for twin pregnancies. See Table S1 for cutoffs.
Abbreviations: EPA, the US Environmental Protection Agency; GWG, gestational weight gain; Hg, mercury; NAM, National Academy of Medicine.

Table S4. Associations between hair Hg concentrations and GWG among women with singleton pregnancy (n=90)

Hair Hg concentrations, ppm	Model 1[a]	Model 2[b]	Model 3[c]
**Total GWG, kg**			
Tertiles (range)			
T1 (≤0.37 ppm)	Ref.	Ref.	Ref.
T2 (0.37-1.01 ppm)	-0.78 (-3.07, 1.51)	-0.22 (-2.63, 2.18)	-0.44 (-2.90, 2.01)
T3 (1.01-4.47 ppm)	-1.94 (-4.23, 0.35)	0.44 (-2.16, 3.05)	0.19 (-2.48, 2.85)
P for trend	0.10	0.77	0.92
EPA reference level			
<1 ppm	Ref.	Ref.	Ref.
≥1 ppm	-1.94 (-3.88, 0.01)	0.23 (-2.10, 2.55)	0.13 (-2.21, 2.47)
P value	0.05	0.85	0.91
**GWG outside the IOM-recommended range**			
Tertiles (range)			
T1 (≤0.37 ppm)	Ref.	Ref.	Ref.
T2 (0.37-1.01 ppm)	0.73 (0.23, 2.20)	0.63 (0.14, 2.64)	0.71 (0.16, 3.05)
T3 (1.01-4.47 ppm)	0.73 (0.23, 2.20)	0.53 (0.12, 2.17)	0.58 (0.13, 2.50)
P for trend	0.57	0.37	0.46
EPA reference level			
<1 ppm	Ref.	Ref.	Ref.
≥1 ppm	0.92 (0.36, 2.40)	0.67 (0.18, 2.42)	0.70 (0.18, 2.60)
P value	0.86	0.54	0.59
**GWG below the IOM-recommended range**			
Tertiles (range)			
T1 (≤0.37 ppm)	Ref.	Ref.	Ref.
T2 (0.37-1.01 ppm)	0.55 (0.15, 1.90)	0.36 (0.06, 1.87)	0.42 (0.07, 2.29)
T3 (1.01-4.47 ppm)	1.59 (0.53, 4.91)	0.45 (0.09, 2.00)	0.56 (0.11, 2.72)
P for trend	0.42	0.30	0.47
EPA reference level			
<1 ppm	Ref.	Ref.	Ref.
≥1 ppm	2.47 (0.94, 6.55)	0.9 (0.22, 3.44)	1.03 (0.24, 4.27)
P value	0.06	0.88	0.96
**GWG above the IOM-recommended range**			
Tertiles (range)			
T1 (≤0.37 ppm)	Ref.	Ref.	Ref.
T2 (0.37-1.01 ppm)	1.14 (0.41, 3.17)	1.43 (0.36, 5.92)	1.42 (0.34, 6.13)
T3 (1.01-4.47 ppm)	0.49 (0.17, 1.40)	0.85 (0.17, 3.92)	0.84 (0.17, 4.00)
P for trend	0.21	0.92	0.89
EPA reference level			
<1 ppm	Ref.	Ref.	Ref.
≥1 ppm	0.42 (0.16, 1.05)	0.58 (0.13, 2.31)	0.58 (0.13, 2.31)
P value	0.07	0.45	0.45

[a] Unadjusted model
[b] Adjusted for age (years), ancestry (White or other), educational attainment (graduate degree or less), pre-pregnancy body mass index (kg/m^2), smoking status (ever or never), total physical activity (hours/week), infertility diagnosis at enrollment (female factor, male factor, or unexplained), plurality (singleton or twins), gestational age at first-trimester weight measurement (weeks), and gestational age at delivery (weeks).
[c] Further adjusted for serum omega-3 long-chain polyunsaturated fatty acid levels based on Model 2.
Abbreviations: EPA, the US Environmental Protection Agency; GWG, gestational weight gain; Hg, mercury; NAM, National Academy of Medicine.

Table S5. Joint associations of hair Hg and serum EPA+DHA with GWG

	**Serum EPA+DHA Tertile (Range, % of Total Fatty Acids)**		
	T1 (1.4-2.6%)	T2 (2.6-3.9%)	T3 (3.9-22.8%)
**Hair Hg Tertile**			
T1 (≤0.40 ppm)	Ref.	1.43 (-1.85, 4.70)	-2.49 (-6.34, 1.37)
T2 (0.40-0.98 ppm)	-2.29 (-5.68, 1.09)	0.45 (-2.99, 3.88)	-0.03 (-3.31, 3.24)
T3 (0.98-4.47 ppm)	-1.70 (-5.45, 2.05)	-0.22 (-3.63, 3.19)	-0.04 (-3.06, 2.98)
**Hair Hg concentration**			
<1 ppm	Ref.	1.92 (-0.47, 4.32)	0.16 (-2.34, 2.66)
≥1 ppm	-0.87 (-4.42, 2.67)	0.54 (-2.67, 3.74)	0.64 (-2.16, 3.44)

Models were adjusted for age (years), ancestry (White or other), educational attainment (graduate degree or less), pre-pregnancy body mass index (kg/m^2), smoking status (ever or never), total physical activity (hours/week), infertility diagnosis at enrollment (female factor, male factor, or unexplained), plurality (singleton or twins), gestational age at first-trimester weight measurement, and at delivery (weeks). The lowest group for both exposures served as the reference. Abbreviations: DHA, docosahexaenoic acid; EPA, eicosapentaenoic acid; Hg, mercury; GWG, gestational weight gain.

Table S6. Stratified analysis of associations between hair Hg concentrations and GWG (without adjustment for serum EPA+DHA levels)[a]

	N	Hair Hg concentration						
		Tertile 1 (lowest)	Tertile 2	Tertile 3 (highest)	*P* for trend	<1 ppm	≥1 ppm	*P* value
**Pre-pregnancy BMI**[b]								
<25 kg/m^2	68	Ref.	0.85 (-1.40, 3.09)	-0.26 (-2.26, 1.74)	0.84	Ref.	-0.58 (-2.39, 1.23)	0.53
≥25 kg/m^2	52	Ref.	-2.37 (-6.17, 1.43)	-1.55 (-5.69, 2.59)	0.38	Ref.	-0.45 (-4.28, 3.37)	0.81
**Number of babies**								
Singleton	90	Ref.	-0.05 (-2.48, 2.37)	0.35 (-2.18, 2.88)	0.81	Ref.	0.20 (-2.11, 2.52)	0.86
Twins	30	Ref.	-3.76 (-9.42, 1.91)	-2.48 (-7.96, 3.00)	0.36	Ref.	-0.56 (-5.32, 4.20)	0.81
**Maternal education**								
Less than graduate degree	54	Ref.	-1.92 (-5.37, 1.52)	-1.77 (-5.62, 2.09)	0.29	Ref.	-0.94 (-4.60, 2.71)	0.60
Graduate degree	66	Ref.	1.20 (-1.65, 4.05)	0.20 (-2.33, 2.73)	0.84	Ref.	-0.26 (-2.54, 2.02)	0.82

[a] Models were adjusted for age (years), ancestry (White or other), educational attainment (graduate degree or less), pre-pregnancy BMI (kg/m^2), smoking status (ever or never), total physical activity (hours/week), infertility diagnosis at enrollment (female factor, male factor, or unexplained), plurality (singleton or twins), gestational age at first-trimester weight measurement (weeks), and gestational age at delivery (weeks), excluding the stratifying variable.
[b] Further adjusted for pre-pregnancy BMI (continuous).
Abbreviations: BMI, body mass index; DHA, docosahexaenoic acid; EPA, eicosapentaenoic acid; GWG, gestational weight gain; Hg, mercury.

Table S7. Associations between hair Hg concentrations and GWG among participants with lower education level (n=42)

	Hair Hg concentration						
	Tertile 1 (lowest)	Tertile 2	Tertile 3 (highest)	*P* for trend	<1 ppm	≥1 ppm	*P* value
Multivariable-adjusted model[a]	Ref.	-1.78 (-5.78, 2.21)	-1.85 (-6.75, 3.05)	0.38	Ref.	-0.94 (-5.49, 3.61)	0.68
Multivariable-adjusted model[a] + serum EPA+DHA levels	Ref.	-2.55 (-6.33, 1.24)	-3.88 (-8.78, 1.03)	0.09	Ref.	-2.11 (-6.61, 2.39)	0.35

[a] Adjusted for age (years), ancestry (White or other), pre-pregnancy body mass index (kg/m^2), smoking status (ever or never), total physical activity (hours/week), infertility diagnosis at enrollment (female factor, male factor, or unexplained), plurality (singleton or twins), gestational age at first-trimester weight measurement (weeks), and gestational age at delivery (weeks).
Abbreviations: DHA, docosahexaenoic acid; EPA, eicosapentaenoic acid; GWG, gestational weight gain; Hg, mercury.

Table S8. Spearman correlations of biomarkers among women with different educational attainment

	Hg	EPA	DHA	EPA+DHA
**Less than graduate degree[a]**				
**Hg**	1	0.25	0.39	0.36
P-value		*0.07*	*0.004*	*0.008*
**EPA**	-	1	0.53	0.70
P-value			*<0.001*	*<0.001*
**DHA**	-	-	1	0.96
P-value				*<0.001*
**EPA+DHA**	-	-	-	1
**Graduate degree**				
**Hg**	1	0.06	0.25	0.18
P-value		*0.66*	*0.04*	*0.15*
**EPA**	-	1	0.67	0.84
P-value			*<0.001*	*<0.001*
**DHA**	-	-	1	0.95
P-value				*<0.001*
**EPA+DHA**	-	-	-	1

Abbreviations: DHA, docosahexaenoic acid; EPA, eicosapentaenoic acid; Hg, mercury.

[a] Including participants without a graduate degree (n=42) or with missing education data (n=12).
